# The Brief negative Symptom Scale (BNSS): a systematic review of measurement properties

**DOI:** 10.1038/s41537-023-00380-x

**Published:** 2023-07-27

**Authors:** Lucia Weigel, Sophia Wehr, Silvana Galderisi, Armida Mucci, John Davis, Giulia Maria Giordano, Stefan Leucht

**Affiliations:** 1 Department of Psychiatry and Psychotherapy, School Of Medicine, Technical University of Munich, Klinikum rechts der Isar, Ismaningerstrasse 22, 81675 Munich, Germany; 2 Department of Mental and Physical Health and Preventive Medicine, University of Campania Luigi Vanvitelli, Largo Madonna delle Grazie 1, 80138 Naples, Italy; 3 Psychiatric Institute, University of Illinois at Chicago (mc 912), 1601 W. Taylor St., Chicago, Il 60612, and Maryland Psychiatric Research Center, Baltimore, MD USA

**Keywords:** Schizophrenia, Human behaviour

## Abstract

**Background:**

Negative symptoms of schizophrenia are linked with poor functioning and quality of life. Therefore, appropriate measurement tools to assess negative symptoms are needed. The NIMH-MATRICS Consensus defined five domains for negative symptoms, which The Brief Negative Symptom Scale (BNSS) covers.

**Methods:**

We used the COSMIN guidelines for systematic reviews to evaluate the quality of psychometric data of the BNSS scale as a Clinician-Rated Outcome Measure (ClinROM).

**Results:**

The search strategy resulted in the inclusion of 17 articles. When using the risk of bias checklist, there was a generally good quality in reporting of structural validity and hypothesis testing. Internal consistency, reliability and cross-cultural validity were of poorer quality. ClinROM development and content validity showed inadequate results. According to the updated criteria of good measurement properties, structural validity, internal consistency and interrater reliability showed good results, while hypothesis testing showed poorer results. Cross-cultural validity and test-retest reliability were indeterminate. The updated GRADE approach resulted in a moderate grade.

**Conclusions:**

We can potentially recommend the use of the BNSS as a concise tool to rate negative symptoms. Due to weaknesses in certain domains further validations are warranted.

## Introduction

Schizophrenia consists of several symptom constructs like general psychopathology, positive and negative symptoms. Positive symptoms, e.g. hallucinations or delusions, are mandatory for the diagnosis and respond well to treatment with antipsychotics while negative symptoms are much harder to treat and are linked with poor functioning and quality of life^1–5^. Therefore, they are of great relevance for treatment of patients with schizophrenia.

For a long time, there was no standardized definition of negative symptoms, which however is needed to be able to assess them and develop treatment options. In January 2005 the NIMH-MATRICS Consensus^6^ took place to review the understanding of negative symptoms and find a more homogeneous definition. The experts involved in the Consensus conference agreed on five domains of the negative symptoms: blunted affect (reduction in emotional expression), alogia (reduction in spoken words and spontaneous elaboration), asociality (decrease in social interaction due to reduction in the drive to engage in relationships), anhedonia (reduction in experience of pleasure for current events or for future anticipated activities) and avolition (reduction in the ability to initiate and persist in goal-directed activities, due to a lack of motivation)^5^.

Different exploratory factor analytic studies, using different tools, supported the two-dimensional model of negative symptoms in subjects with schizophrenia. According to this model, avolition, anhedonia, and asociality constitute the Motivational Deficit domain (MAP), while blunted affect and alogia the Expressive Deficit domain (EXP)^5^. This model is supported by the evidence that the two domains are related to different behavioral and neurobiological features, as well as different clinical and social outcomes^7^. However, more recently, multicenter confirmatory factor analyses have questioned the validity of the two-factor solution and suggested that a five-factor model or a hierarchical model (five negative symptoms as first-order factors and the two domains, MAP and EXP, as second-order factors) better fit the data, irrespective of the assessment scale, sample nationality/language or stage of illness^8,9^.

There are many scales in schizophrenia that try to assess negative symptoms; however, they do not cover the 5 domains defined by the NIMH^6^ as most of them have been developed years before the Consensus. Therefore, the experts involved envisaged the need to develop new assessment tools. The „Clinical Assessment Interview for Negative Symptoms (CAINS)”^10–12^ was initially developed to be a quite long scale, covering the 5 domains in extensive detail but requiring more time for the assessment. For the other scale the experts concentrated on creating a more concise instrument which would be suitable for a widespread use in clinical trials, and proposed "The Brief Negative Symptom Scale (BNSS)"^13^. The BNSS consists of 13 items, which are divided into 6 subscales: 1. Anhedonia, 2. Distress, 3. Asocialty, 4. Avolition, 5. Blunted affect, 6. Alogia. It is based on a semi structured interview and rated on a 7-point scale from 0 (absent) to 6 (severe). The administration takes about 15 minutes. A total score is calculated by summing all 13 items, possible scores can range from 0 to 78 points.

As there has not been an attempt to systematically review the psychometric properties of existing negative symptom scales, our aim was to evaluate the quality of the BNSS by applying the COnsensus-based Standards for the selection of health Measurement INstruments (COSMIN)^14–16^ guidelines for systematic reviews of patient-reported outcome measures.

## Methods

The methods used in this systematic review follow the guidelines described by Prinsen et al., 2018: COSMIN guideline for systematic review of patient-reported outcome measures^14–16^. They were developed to objectively evaluate rating scales in a standardized way and include several steps: evaluate the methodological quality of the included studies by using the COSMIN Risk of Bias checklist, apply criteria for good measurement properties and grade the quality of the evidence by using the modified GRADE approach according to COSMIN.

The COSMIN methodology was primarily created for patient-rated outcome measures (PROMs), however the methodology can be adapted and used on clinician-reported outcome measures (ClinROMs) which is the category the Brief Negative Symptom Scale falls into^14–17^.

### Literature search strategy for validation studies

Two reviewers (LW and SW) independently performed a literature search by searching the databases PubMed and Web of Science for journal articles published in English between January 2010 and June 2022 inclusive, disagreements were resolved by finding consensus, if needed by a third reviewer (SL). The search terms used were “BNSS” OR “Brief Negative Symptom Scale”.

### Evaluation of measurement properties

The evaluation of the measurement properties was independently performed by two reviewers (LW and SW) for all the following steps. If any disagreements became apparent, a consensus was reached by consulting a third, professor-level reviewer (SL).

### Assessing the risk of bias

The Risk of Bias Checklist^14–16^ was developed to rate the reporting quality of studies for specific criteria.

The standards for good methodological quality are sorted by criteria in 10 boxes: ClinROM development, content validity, structural validity, internal consistency, cross-cultural validity/measurement invariance, reliability, measurement error, criterion validity, hypothesis testing for construct validity, responsiveness.

Each measurement property is scored on a four‐point scale using the descriptors “very good”, “adequate”, “doubtful”, and “inadequate”. A “not applicable” option is also included for each property. An overall score for the methodological quality of each measurement property is determined by taking the lowest rating of any of the items in a box, which is called "worst score counts” principle.

The first two boxes of the Risk of Bias checklist, “outcome measure tool development” and “content validity” which relate to content validity, were deemed to be applicable to only the original publication which describes the development of the scale.

Criterion validity and responsiveness were excluded from this systematic review because there is no true gold standard for negative symptom assessment scales. Even the most frequently used scale in schizophrenia, the Positive and Negative Syndrome Scale (PANSS)^18^, has not undergone all steps required by the COSMIN criteria including the evaluation of content validity. Therefore, it can’t serve as a true gold standard.

For methodological details please refer to the following document on the COSMIN website: https://cosmin.nl/wp-content/uploads/COSMIN_risk-of-bias-checklist_dec-2017.pdf).

### Assessing the updated criteria for good measurement properties

The quality of the instrument itself was assessed by using the updated criteria for good measurement properties^14–16^, which comprise eight criteria: structural validity (i.e., the scale validity assessed by using Rasch analysis/Item Response Theory or Classical Test Theory), internal consistency (measured by the Cronbach’s alpha when at least low evidence of structural validity is available), reliability (inter-rater or test-retest reliability, measured by intraclass correlation coefficient), measurement error (determining the limits of agreement and smallest detectable change against a measure of the minimal important change), hypotheses testing for construct validity (assessing whether a clear hypothesis was defined and tested), cross-cultural validity/ measurement invariance (i.e., measurement invariance across groups defined by ethnicity or age/ gender), criterion validity and responsiveness (measured as correlation with gold standard or area under the curve ≥ 0.70). Criterion validity and responsiveness could not be evaluated due to the lack of gold standards, as mentioned above.

### Grading the quality of evidence

The grade approach was used to grade the quality of evidence which refers to the confidence that the result is trustworthy. It is based on the Grading of Recommendations Assessment, Development, and Evaluation (GRADE) approach for systematic reviews of clinical trials, modified by the COSMIN group^14–16^ and uses four factors to determine the quality of the evidence: risk of bias (quality of the studies), inconsistency (of the results of the studies), imprecision (total sample size of all included studies) and indirectness (evidence comes from different populations, interventions or outcomes than the population of interest in the review). The quality of the evidence is graded as high, moderate, low or very low. The starting point is always the assumption that the evidence is of high quality and is subsequently downgraded by one, two or three levels per factor if the criteria are not sufficient (see Table 1).

**Table 1:** Definitions of GRADE according to COSMIN.

Quality of evidence	Lower if (either/or)
High = We are very confident that the true measurement property lies close to that of the estimate of the measurement property	*Risk of Bias* -1 Serious -2 Very serious -3 Extremely serious
Moderate = We are moderately confident in the measurement property estimate: the true measurement property is likely to be close to the estimate of the measurement property, but there is a possibility that it is substantially different	*Inconsistency* -1 Serious -2 Very serious
Low = Our confidence in the measurement property estimate is limited: the true measurement property may be substantially different from the estimate of the measurement property	*Imprecision* -1 total *n* = 50–100 -2 total *n* < 50
Very low = We have very little confidence in the measurement property estimate: the true measurement property is likely to be substantially different from the estimate of the measurement property	*Indirectness* -1 Serious -2 Very serious

#### Risk of bias

To use the risk of bias assessment for the GRADE approach, each risk of bias item/box was evaluated with applying criteria from Table 2. Following the worst-case approach, if one Risk of Bias item/box has an extremely serious risk of bias it can be downgraded by three points. Only if the given item had a determinate result in Step 2 “updated criteria of good measurement” (received a “+” or “-“ rating and not a “?”), it was considered to downgrade the confidence in the evidence of the item.

**Table 2:** GRADE downgrading criteria for risk of bias.

Risk of Bias	Downgrading for Risk of Bias
No	There are multiple studies of at least adequate quality, or there is one study of very good quality available
Serious	There are multiple studies of doubtful quality available, or there is only one study of adequate quality
Very serious	There are multiple studies of inadequate quality, or there is only one study of doubtful quality available
Extremely serious	There is only one study of inadequate quality available

#### Inconsistency

As we didn’t quantitatively pool (meta-analyzed) the results, our criteria to downgrade was as follows: if no inconsistency was found the scale was not downgraded, if little inconsistency was found with valid explanation the scale was not downgraded, if little inconsistency was found with no explanation or moderate to high inconsistency was found with a valid explanation for these results we downgraded -1 (serious), if a moderate to high inconsistency was found with no satisfactory explanation, we downgraded -2 (very serious).

#### Imprecision

This evaluates the total sample size of all included studies. If the sample size was *n* = 50–100 we downgraded -1, if the sample size was *n* < 50 we downgraded −2.

#### Indirectness

There was a downgrading for indirectness if the patients included in the studies were not part of the population of interest. For this review, the sample groups must consist of patients with schizophrenia or schizoaffective disorder.

If there was a comparator group of patients with a different disease or a healthy control group, no downgrade was given.

### Retrospective re-validation

The authors of two of our included validation studies^19,20^, AM and SG, who also participated as co-authors in this systematic review, re-validated structural validity for one and internal consistency for both studies (see supplement).

## Results

### Literature search strategy for validation studies

**Fig. 1:**
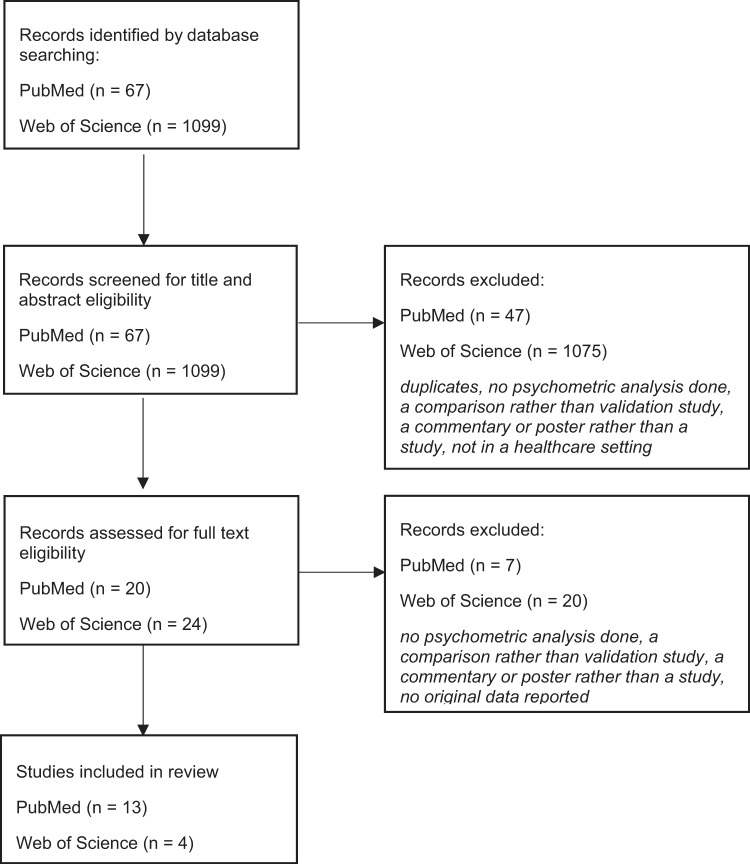
Flowchart. Literature search PubMed and Web of Science.

A total of sixty-seven articles (*n* = 67) were found on PubMed, twenty articles (*n* = 20) were chosen by title/abstract and thirteen of these articles (*n* = 13) were included in the systematic review. A total of one thousand ninety-nine articles (*n* = 1099) were found on Web of Science, twenty-four (*n* = 24) were chosen by title/abstract and four (*n* = 4) were included in the systematic review. The literature search is shown in the Flowchart in Fig. 1. The general characteristics of the included studies are portrayed in Table 3.

**Table 3:** General characteristics of included validation studies.

Study	Language	Country	Population	Mean (SD) age	Gender (% female)	Number
Kirkpatrick, 2010^9^	English	United States	Patients with schizophrenia	48.1 (6.6)	20%	20
Strauss, 2012^16^	English	United States	Patients with schizophrenia or schizoaffective disorder	42.1 (11.8)	25.3%	146
Strauss, 2012^20^	English	United States	Patients with schizophrenia or schizoaffective disorder	42.2 (11.1)	26%	100
Mane’, 2014^21^	Spanish	Spain	Outpatients with schizophrenia	37.34 (11.71)	30%	20
Mucci, 2015^17^	Italian	Italy	Patients with schizophrenia	40.1 (10.7)	30.2%	912
Strauss, 2015^22^	English	United States	Patients with schizophrenia	40.8 (12.5)	46%	50
			Patients with bipolar disorder	38.9 (12.7)	63%	46
			Healthy controls	36.7 (15.3)	52%	27
Bischof, 2016^23^	German	Switzerland	In -/Outpatients with schizophrenia or schizoaffective disorder	31.5 (10.9)	25.3%	75
Polat Nazlı, 2016^26^	Turkish	Turkey	In -/Outpatients with schizophrenia	34.6 (8.3)	24%	75
Virgulino de Medeiros, 2018^18^	Brazilian Portuguese	Brazil	Outpatients with schizophrenia	39.5 (12)	29%	111
Gehr, 2019^24^	Danish	Denmark	In -/Outpatients with schizophrenia or schizoaffective disorder	33.1 (10.8)	34.7%	49
Mucci, 2019^13^	Multiple	Austria, Czech Republic, Denmark, France, Italy, Norway, Poland, Switzerland, Russia, Turkey	In -/Outpatients with schizophrenia	37.3 (11.3)	36.5%	249
Wojciak, 2019^19^	Polish	Poland	Patients with paranoid schizophrenia	44 (13)	50%	40
San Ang, 2019^14^	English	Singapore	Patients with paranoid schizophrenia	40.42 (10.17)	44.53%	274
Hashimoto, 2019^27^	Japanese	Japan	In -/Outpatients with schizophrenia	37.9 (9.7)	40%	10
Jeakal, 2020^15^	Korean	Korea	Patients with paranoid schizophrenia	41.91 (11.01)	49.7%	173
Seelen-de Lang, 2020^33^	Dutch	Netherlands	Patients with schizophrenia or schizoaffective disorder or psychotic disorder	44.8 (13.6)	21,4%	28
Sun, 2021^23^	Chinese	China	Patients with schizophrenia	51	43%	149

### Assessing the risk of bias

#### Content validity

##### ClinROM development

ClinROM development is per definition not a measurement property, it is however considered when evaluating content validity. It asks about the general design requirements and if the assessment of comprehensibility and comprehensiveness during pilot testing was performed.

One study^13^ was evaluated for the ClinROM development and received an “inadequate” rating because it is not clear if the patients were asked about comprehensibility or comprehensiveness of the scale (see Table 4).

**Table 4:** Cosmin risk of bias and updated criteria of good measurement results.

Measurement property (No. of studies assessing measurement property)	Cosmin Risk of Bias				Updated Criteria of good measurement		
	Very good	Adequate	Doubtful	Inadequate	+	−	?
ClinROM Development (*n* = 1)^a^				1	NA	NA	NA
Content validity (*n* = 0)^a^					NA	NA	NA
Structural validity (*n* = 10)	5	2	1	2	5	0	5
Internal consistency (*n* = 15)	5	0	0	10	4	0	11
Cross-cultural validity (*n* = 1)	0	0	1	0	0	0	1
Interrater reliability (*n* = 11)	0	3	8	0	8	2	1
Test-retest reliability (*n* = 5)	0	0	5	0	0	0	5
Hypotheses testing for construct validity ­*convergent validity* (*n* = 16)	6	8	0	2	10	6	0
Hypotheses testing for construct validity ­*discriminant validity* (*n* = 15)	6	7	0	2	5	10	0

^a^ClinROM Development and Content validity are only applicable to the development publications of the scale

##### Content validity

A content validity study refers to a study asking patients and professionals about the relevance, comprehensiveness, or comprehensibility of an existing ClinROM. Such a study can be performed by the developers or by researchers who were not included in the initial development.

No information was given if testing on content validity was performed, therefore it could not be considered in this systematic review.

#### Internal structure

##### Structural Validity

Structural validity measures the degree to which the scores of the scale are an adequate reflection of the construct to be measured. Therefore, it is only relevant if the scale is based on a reflective model, where it is assumed that all items in a scale or subscale are manifestations of one underlying construct and are expected to be correlated. This means that each item and subscale of the BNSS measure the same underlying construct which is negative symptoms in patients with schizophrenia or schizoaffective disorder.

Structural validity is measured by performing factor analysis. Confirmatory factor analysis is preferred, which results in a “very good“ rating while studies with exploratory factor analysis only receive an “adequate“ rating.

Of the overall seventeen included studies, ten performed a factor analysis. Five^19–23^ performed a confirmatory factor analysis which resulted in a “very good” rating, two “adequate” ratings^24,25^ for only performing exploratory factor analysis, one “doubtful”^26^ rating for exploratory factor analysis compared with a sample size < 100 and two “inadequate”^13,27^ ratings also due to an inadequate sample size (see Table 4).

##### Internal Consistency

Fifteen papers reported on internal consistency, five^19–21,23,28^ received a “very good” rating. The remaining ten^13,22,25–27,29–33^ received an “inadequate” as Cronbach’s alpha was only reported for the overall scale and not for the subscales individually (see Table 4).

##### Cross-cultural validity/ Measurement invariance

One study^31^ reported on cross-cultural validity by comparing patients with schizophrenia, bipolar patients and a healthy control group with each other. The reporting quality of the validation received a “doubtful” rating (see Table 4).

##### Remaining measurement properties

Reliability: Eleven papers reported on interrater reliability. Three papers^23,32,33^ were rated “adequate” and the remaining eight^13,19,22,25–27,30,34^ received a “doubtful” rating due to an inappropriate time interval or missing information on the rating conditions and the similarity of instructions, administrations, environment etc. Five papers^13,23,27,29,30^ also tested for test-retest reliability. None of them however calculated ICCs for the test-retest reliability, but only Pearson’s correlations. The use of Pearson’s or Spearman’s correlations is considered doubtful due to the COSMIN methodology and therefore leads to an indeterminate result later on (see Table 4).

##### Hypotheses testing for construct validity

Convergent validity: Hypotheses testing for convergent validity assumes that the investigated scale is valid for the construct it’s supposed to measure. It is examined by comparing it with another scale that measures the same or similar construct.

Ideally the comparator tool has very good measurement properties and measure the identical construct. However, this turned out to be difficult to evaluate as we are simultaneously rating the measurement properties for other existing negative symptom scales^35^ and yet there is no available data on their overall measurement properties. Additionally, due to the construct of negative symptoms going through many changes over the past decades, only similar constructs could be found to be compared but not identical ones.

Sixteen papers reported on convergent validity. Six^20,21,23,25,32,34^ received a “very good”, eight^13,19,26–31^ received an “adequate” and two^22,33^ an “inadequate” rating because they failed to be clear about what construct the comparator tools measure (see Table 4).

Discriminant validity: Hypotheses testing for discriminant validity assumes that the investigated scale is valid for the construct it wants to measure and compares it to another scale that measures a different construct. Mostly positive symptom scales were used as a discriminant construct as well as depression scales as it is of great importance to differentiate between symptoms of depression and negative symptoms.

Fifteen papers reported on discriminant validity. Six^20,21,23,25,32,34^ received a “very good” and seven^13,19,26,27,29–31^ an “adequate” rating. Two^22,33^ studies received an “inadequate” as their rating as they failed to be clear about what construct the comparator tools measure (see Table 4).

### Assessing the updated criteria for good measurement properties

#### Internal structure

##### Structural validity

Although ten studies performed a factor analysis, five^13,24–27^ are indeterminate and received a “?” due to missing calculations. This is inconvenient as all five validated the two-factor structure of the BNSS with a MAP and EXP subscale.

The remaining five studies^19–23^ all had sufficient results and therefor received “+” ratings (see Table 4).

In both their validation studies, Mucci et al. ^19,20^. found sufficient results for the five-factor model and the hierarchical model with CFI > 0.95. It needs to be stated that they excluded the Distress item in their analyses as it is not an original domain named by the NIMH-MATRICS Consensus^5^. Jeakal et al. ^22^ favored the five-factor model with TLI and CFI resulting in numbers > 0.95 for the five-factor as well as the 2nd order five-factor hierarchical model. Sun et al. ^23^ also favored the five-factor model with a CFI of 0.996 and TLI of 0.999 but had results of > 0.97 for CFI and TFI for all their tested models.

Ang et al. ^21^ had sufficient results for all their tested factor structures with TLI and CFI > 0.95. The second-order model, where the Distress item was excluded, had the highest results with a CFI = 0.999. They named the five domains as first-order factors and Emotional Expressivity and Motivation/Pleasure as second-order factors.

Overall, it can be said that the hierarchical model and the five-factor model show the best results in the included studies and no clear recommendation can be given on which model should be used.

##### Internal consistency

Four studies^19–21,23^ calculated Cronbach’s alpha for the individual subscales and received a “+” rating with Cronbach’s alpha ranging from 0.8 to 0.97 for their subscales. One^22^ study only calculated Cronbach’s alpha if item deleted and no subscale scores. Therefore, it received a “?” as these results are indeterminable. For the remaining ten^13,25–33^ studies that calculated Cronbach’s alpha, the criteria for „at least low evidence for sufficient structural validity“ was not met. Therefore, they all received “?” as their rating. As five studies however have determinable results with Cronbach’s alpha > 0.7 for all subscales, sufficient internal consistency can be assumed (see Table 4).

##### Cross Cultural validity/ Measurement invariance

One study^31^ tested measurement invariance comparing patients with schizophrenia, patients with bipolar disorder and a healthy control group. No statement can be made as the results are indeterminate “?” (see Table 4).

#### Remaining measurement properties

##### Reliability

Eight^13,19,22,23,25,27,30,32^ of the eight studies evaluating the scales’ interrater reliability were sufficient and received a “+” rating, one^26^ was indeterminate “?” and one^34^ was insufficient “−“ due to the Distress item with an ICC of 0.46, while another one^33^ was insufficient due to an ICC of 0.55 for Blunted affect, which isn’t explicable (see Table 4). All other subscales had an ICC > 0.80 for both studies. The range for the intraclass correlation without the Distress item is 0.77–0.98 while the range for the Distress item is 0.46–0.94. The study by Gehr et al. ^34^ received a particularly poor result for the Distress item (ICC = 0.46), the reason being unclear.

##### Hypotheses testing for construct validity

The three hypotheses to be tested according to COSMIN are:

1. Correlations with instruments measuring similar constructs should be ≥ 0.50.
2. Correlations with instruments measuring unrelated constructs should be < 0.30.
3. Correlations defined under 1 and 2 should differ by a minimum of 0.10.

Convergent validity: Sixteen studies tested for convergent validity, ten^13,19,20,23,26–29,31,32^ received a “+” and six^21,22,25,30,33,34^ a “-“ (see Table 4). Convergent validity was calculated using multiple different scales. With the “Scale for the Assessment of Negative Symptoms (SANS)”^36,37^, correlations ranged from 0,44 to 0,95. We decided to exclude the Distress item from this range as it had a correlation as low as −0,11 with the SANS total. “The Positive and Negative Syndrome Scale (PANSS)”^18^ negative subscale has correlations ranging from 0,31 to 0,9 and “the Brief Psychiatric Rating Scale (BPRS)”^38^ negative subscale resulted in correlations ranging from 0,1 to 0,87. These three (sub)scales were most used as comparator tools. As the sixteen studies were performed in a wide range of cultures and were also often performed in different languages, a certain inconsistency was expected. The range throughout these studies was however higher than anticipated, with all results ranging between sufficient and insufficient range. One study^22^ measured convergent validity for the total scale correlation between the BNSS and the CAINS and resulted in a correlation of 0.90.

Discriminant validity: Fifteen studies tested for discriminant validity, five^19,20,23,29,33^ received a “+” and ten^13,21,22,25–27,30–32,34^ a “-“ (see Table 4). For discriminant validity, an even greater number of different comparator tools was used, which is why only the most used (sub-)subscales will be mentioned here. The PANSS positive subscale had correlations with the BNSS from −0,13 to 0,49, the PANSS general psychopathology subscales’ correlation ranged from −0,21 to 0,58 and the Hamilton Depression Rating Scale (HDRS) correlation ranged from −0,13 to 0,31. Other (sub-)scales however had only results which were below the hypothesis testing limit of 0,3. For example, the Calgary Depression Scale (CDSS) with a correlation ranging from −0,38–0,28, the BPRS positive subscale with a correlation ranging from −0,31–0,08 and the Young Mania Rating Scale (YMS) with a correlation ranging from −0,1 - (−0,07). The results of discriminant validity are similar to the results of convergent validity in terms of consistency which can also be explained through the cultural differences and multiple different languages of the study groups.

### Grading the quality of evidence

1. Structural validity, internal consistency, interrater reliability, convergent and discriminant validity all had either multiple studies of adequate quality or at least one of very good quality. There was only one study of doubtful quality for cross-cultural validity, however, the result was indeterminate and will therefore not be considered as a criterion for downgrading. The same applies for test-retest reliability where there were only studies of doubtful quality but with indeterminable results. The BNSS scale will therefore not be downgraded for Risk of Bias.
2. Inconsistency was found in convergent and discriminant validity, which is explained in length under “Updated criteria of good measurement” and therefore a downgrade of −1 was proposed. The proposals for downgrading were discussed between the two independent raters and consensus was found with a third professor-level rater to overall give a downgrading of −1 for the scale’s inconsistency as there was sufficient explanation found. This changes the “high” grade to a “moderate” grade.
3. The total included sample size of all studies is *n* = 2554, so there will not be a downgrade for imprecision. The grade for the evidence of quality will therefore stay “moderate”.
4. The tested population only consisted of in-/outpatients with schizophrenia or schizoaffective disorder for all included studies. There is no need to downgrade for indirectness, which results in a “moderate” rating for the BNSS scale.

The overall quality of the evidence is now considered “moderate” for the BNSS scale, which leads to the conclusion that there is moderate quality evidence that the measurement properties of interest are sufficient.

## Discussion

Even though the BNSS^13^ is a relatively new scale, it has been used in many different countries and cultures. As it is a short measurement tool, it is attractive for clinical studies. However, to the authors‘ knowledge, this is the first systematic review to examine the measurement properties of the scale. The evaluation was undertaken using the COSMIN guidelines and the COSMIN Risk of Bias checklist^14–16^. Seventeen studies were identified as relevant by a systematic literature search and included in this study.

The original publication^13^ failed to test for or report on ClinROM development, which includes the general design requirement as well as conducting a cognitive interview study asking patients/professionals about the relevance /comprehensibility/ comprehensiveness of the included items. This must be considered a weakness of the BNSS. However, the content validity of the BNSS is based on the 2005 NIMH Consensus^6^, thus, it would be possible to test the content validity retrospectively. It is of great importance to report or perform the evaluation of ClinROM development and content validity by using the COSMIN Risk of Bias checklist to make the overall results of the validation of the scale more reliable and provide well-reported psychometric data. One possibility would be to retrospectively validate the content validity by forming focus groups, which could potentially improve the recommendability of the scales.

The BNSS demonstrates good psychometric properties for structural validity, internal consistency, reliability and hypothesis testing. However, the quality of evidence for cross-cultural validity is somewhat poorer. Nonetheless, it is of great importance that a rating scale is culturally adaptable, produces comparable results and is an adequate reflection of the original version in different populations, countries and languages. Therefore, cross-cultural validity needs to be properly validated. As the BNSS scale is available in multiple translations, further validation studies should be relatively easy to conduct.

We recommend validating internal consistency according to the COSMIN guideline as currently most studies only calculated internal consistency for the total scale instead of each individual subscale. Such a retrospective re-validation is possible according to COSMIN criteria, and for two of the included studies^19,20^ it improved our rating. It’s equally important to mention that internal consistency can only receive a positive rating if the criteria for “at least low evidence for sufficient structural validity” is met. Therefore, we recommend performing confirmatory factor analysis for the BNSS scale as it would help determine its structural validity and also its internal consistency. Indeed, performing further confirmatory analyses would allow to overcome the limits of the exploratory factor analyses and to replicate more recent findings of a five-factor or a hierarchical model of negative symptoms^8,9^, which were also supported by our post-hoc analysis of the study conducted by Mucci et al. To define the correct characterization of negative symptom structure could have important implications, since the 2-factor structure might have foreclosed the identification of neurobiological bases or therapeutic effects that are specific to one of the five domains. Therefore, considering current findings, future versions of the DSM-5 should consider each of the five domains separately, as described by NIMH-MATRICS Consensus^6^.

The additional Distress item turned out to be a weakness of the BNSS scale as it repeatedly showed poorer results and was already excluded by some of the authors in their validation studies. We therefore recommend revising the scale in this regard and in the future exclude the item from the scale, as it was not part of the original five domains established by the NIMH Consensus^6^.

Based on the results of the evaluation, an overall judgement of the recommendability of the BNSS scale is the final product of the evaluation. According to the COSMIN guidelines^14–16^ ClinROMs are categorized into three categories:

(A) ClinROMs with evidence for sufficient content validity (any level) AND at least low-quality evidence for sufficient internal consistency
(B) ClinROMs categorized not in A or C
(C) ClinROMs with high quality evidence for an insufficient measurement property

ClinROMs categorized as “A" can be recommended for use and results obtained with these ClinROMs can be trusted. ClinROMs categorized as ”B” have potential to be recommended for use, but they require further research to assess the quality of these ClinROMs. ClinROMs categorized as “C” should not be recommended for use.

No testing for sufficient content validity was performed. Due to this reason the BNSS scale is categorized as (B).

However, content validity is defined as the degree to which the content is an adequate reflection of the construct to be measured. The BNSS is based on the NIMH Consensus with the aim of finding a standardized definition of the negative symptom construct. Therefore, it creates adequate content validity for the scales that are based on it. Still, as mentioned above, ClinROM development and content validity need to be evaluated in the future to grow the confidence in the scale.

It needs to be mentioned that this systematic review only evaluated the BNSS scale according to the COSMIN guidelines for systematic reviews. This tool is relatively new and follows rather strict criteria, while other methodologies might reach different conclusions. Most scales to rate patients with schizophrenia would probably receive these or even worse results. In the future the COSMIN guidelines could be used prospectively to create new rating scales or conduct validation studies so that all demanded criteria are included.

Our study has potential limitations. We were not able to perform a metanalysis on this topic as the data were presented in many different ways and therefore quantitively summarizing the results wasn’t possible. Furthermore, no protocol was written during the process.

The BNSS is still recommendable, compared to the older negative symptom scales such as the SANS^36,37^, the BPRS^38^, the “Krawiecka-Manchester-Scale” (KMS)^39^, the “A Negative Symptom Rating Scale” (NSRS)^40^, the PANSS^18^, the “Schedule for the Deficit Syndrome (SDS)”^41^, the “High Royds of Evaluation of Negativity Scale (HEN)”^42^ and the “Negative Symptom Assessment of Chronic Schizophrenia Patients (NSA-16)”^43^. Several of them (BPRS, KMS, NSRS, PANSS) do not cover the five negative symptom domains established by the NIMH Consensus. The remaining scales (SANS, SDS, HEN, NSA-16) showed poorer results for the psychometric properties as evaluated in “Clinician-reported negative symptom scales in schizophrenia: a systematic review of measurement properties.” (LW, SW (joined first authors), SG, AM, JD, SL; *manuscript in preparation*). The only “competitor” of the BNSS scale is the CAINS scale^10–12^ which we examined in a different paper: “Clinical Assessment Interview for Negative Symptoms (CAINS): a systematic review of measurement properties.” (SW, LW, JD, AM, SG, SL; manuscript under review). The CAINS also received a “moderate” rating (manuscript under review), which is why no clear recommendation can be given on which scale is of better quality than the other. As the BNSS however needs a shorter administration time as compared to the CAINS (15 minutes vs. 30 minutes), we would recommend the use of the BNSS over the CAINS if there is a need of a quicker evaluation of negative symptoms. The confidence in both rating scales could still be improved by conducting further validation studies. Moreover, a comparison of the BNSS and the CAINS would be of great interest as they were both developed based on the NIMH Consensus around the same time. So far only one study^22^ has compared the two scales which was restricted to convergent validity.

To conclude, the BNSS performed well regarding structural validity, internal consistency, reliability and hypothesis testing for convergent validity; however, the measure did not attain satisfying results regarding hypothesis testing for discriminant validity and only one study reported on cross-cultural validity. Considering the overall result of this systematic review, we classify the BNSS as a potentially recommendable tool to rate negative symptoms, especially if a quick administration time is needed. Further validation studies including the specific requirements made by COSMIN should however be conducted in order to address the weaknesses of BNSS pointed out in this systematic review to further improve the confidence in this scale.

## Supplementary information

Systematic review BNSS (Weigel et al)_supplemental material

## Data Availability

We do not have individual patient data. All ratings can be found in the tables.
