# Supplementary material for: The Brief negative Symptom Scale (BNSS): a systematic review of measurement properties
Source: Schizophrenia (Heidelb). 2023 Jul 27;9(1):45. doi: 10.1038/s41537-023-00380-x (PMC10374652; doi:10.1038/s41537-023-00380-x)
Supplement: Supplementary file 1 — Systematic review BNSS (Weigel et al)_supplemental material [file 41537_2023_380_MOESM1_ESM.docx]

the Brief negative Symptom Scale (BNSS): a systematic review of measurement properties.

Lucia Weigel, Sophia Wehr, Silvana Galderisi, Armida Mucci, John Davis, Giulia Maria Giordano, Stefan Leucht*

Lucia Weigel ([lucia.weigel@tum.de](about:blank)) Department of Psychiatry and Psychotherapy, School Of Medicine, Technical University of Munich, Klinikum rechts der Isar, Ismaningerstrasse 22, 81675 Munich, Germany, E-Mail: lucia.weigel@tum.de

Sophia Wehr ([sophia.wehr@tum.de](about:blank)) Department of Psychiatry and Psychotherapy, School Of Medicine, Technical University of Munich, Klinikum rechts der Isar, Ismaningerstrasse 22, 81675 Munich, Germany, E-Mail: [sophia.wehr@tum.de](about:blank)

Silvana Galderisi Department of Mental and Physical Health and Preventive Medicine, University of Campania Luigi Vanvitelli, Largo Madonna delle Grazie 1, 80138 Naples, Italy, E-Mail: silvana.galderisi@gmail.com

Armida Mucci Department of Mental and Physical Health and Preventive Medicine, University of Campania Luigi Vanvitelli, Largo Madonna delle Grazie 1, 80138 Naples, Italy, E-Mail: armida.mucci@gmail.com

Prof. John M. Davis, MD (davisjm@uic.edu): Psychiatric Institute, University of Illinois at Chicago (mc 912), 1601 W. Taylor St., Chicago, Il 60612, and Maryland Psychiatric Research Center, Baltimore, MD, USA

Giulia Maria Giordano Department of Mental and Physical Health and Preventive Medicine, University of Campania Luigi Vanvitelli, Largo Madonna delle Grazie 1, 80138 Naples, Italy, E-Mail: [giuliamgiordano@gmail.com](mailto:giuliamgiordano@gmail.com)

*Corresponding Author:

Prof. Stefan Leucht, MD (stefan.leucht@tum.de) Department of Psychiatry and Psychotherapy, School of Medicine, Technical University of Munich, Klinikum rechts der Isar, Ismaningerstrasse 22, 81675 Munich, Germany, Tel: +49-89-4140-4249, Fax: +49-89-4140-4888, E-Mail: stefan.leucht@tum.de; And Institute of Psychiatry, Psychology and Neuroscience, King’s College London, Department of Psychosis Studies

**Index**

**1.**

| **Confirmatory Factor analysis and internal consistency of the Brief Negative Symptom Scale (BNSS) in a large sample of Italian patients with schizophrenia** | **Page 3** |
| --- | --- |
| **Methods** | **Pages 3-4** |
| **Results** | **Page 4** |
| **Table S1** | **Page 5** |
| **Table S2** | **Page 6** |

**2.**

| **Internal consistency of the Brief Negative Symptom Scale (BNSS) in a large European, multicenter study** | **Page 7** |
| --- | --- |
| **Methods** | **Page 7** |
| **Results** | **Page 7** |
| **Table S3** | **Page 8** |

| **References** | **Page 9** |
| --- | --- |

**1. Confirmatory Factor analysis and internal consistency of the Brief Negative Symptom Scale (BNSS) in a large sample of Italian patients with schizophrenia**

**1.1 Methods**

**Participants**

The data set used to perform the confirmatory factor analysis of the Brief Negative Symptom Scale in the current investigation was that included in the study of Mucci et al., 2015 [1].

**Factor structure and internal consistency of the BNSS**

The factor structure of the BNSS was examined using a confirmatory factor analysis (CFA), in order to evaluate the fit of 4 models of the latent structure of negative symptoms. The first model (unidimensional model) evaluated whether all five domains (avolition, anhedonia, asociality, blunted affect and alogia) reflected a single negative symptom structure; the second model considered the two negative symptom dimensions: Motivation Deficit (MAP) and Expressive Deficit (EXP); the third model tested the 5-factor model of negative symptoms (each negative symptom domain constituted a factor); finally the fourth model (hierarchical model) was designed with 5 first-order factors (five negative symptom domains) and 2 second-order factors (MAP and EXP). In all the CFA models the item “Lack of normal distress” was not included since it is not considered a negative symptom according to the current conceptualization of negative symptoms [2] and since the prior exploratory factor analysis conducted in the same sample [1] reported low communalities for this item.

To assess the global fit, the following indices were applied: χ2 value, the Akaike information criterion (AIC), the sample size-adjusted Bayesian information criterion (BIC), the Tucker Lewis index (TLI), the comparative fit index (CFI), the root mean square error of approximation (RMSEA) and the standardized root mean square residual (SRMR). The information criteria, AIC and BIC, are relative fit indices of model parsimony that take into account model complexity based on degrees of freedom. Lower values indicate better model fit. A good fit included a χ2 value not statistically significant, lower BIC and AIC values, TLI and CFI values of at least 0.95, RMSEA and SRMR no greater than 0.08.

Internal consistency of the BNSS, its subscales and factors was assessed using the Cronbach’s alpha value.

All the analyses were carried out with SPSS Statistics and AMOS (both versions 28).

**1.2 Results**

**CFA analysis**

Results of the CFA analyses are reported in Table S1. The 1-factor and 2-factor models provided poor fit, while the 5- factor model and the hierarchical model provided the best fit.

**BNSS internal consistency**

Results regarding the BNSS internal consistency are reported in table S2.

The 13-item BNSS had good internal consistency (Cronbach's alpha = 0.96) and there were no changes in Cronbach's alpha after removing Distress. Cronbach's alpha values of sub-scales ranged from 0.88 to 0.96. For MAP and EXP factors, Cronbach's alpha was 0.94 and 0.95 respectively.

| Model | X^2^ Value (df) | AIC | BIC | TLI | CFI | RMSEA | SRMR |
| --- | --- | --- | --- | --- | --- | --- | --- |
| 1 Factor | 4096.68 (54) | 4144.68 | 4145.37 | 0.614 | 0.684 | 0.287 | 0.09 |
| 2 Factor | 2205.22 (53) | 2255.22 | 2375.61 | 0.791 | 0.832 | 0.211 | 0.08 |
| 5 Factor | 195.174 (44) | 263.17 | 426.91 | 0.982 | 0.988 | 0.061 | 0.01 |
| Hierarchical | 211.192 (48) | 271.19 | 415.66 | 0.982 | 0.987 | 0.061 | 0.02 |

**Table S1.** Model fit results from CFA on negative symptoms as assessed by the Brief Negative Symptom Scale (BNSS) (N=912).

CFA = confirmatory factor analysis; AIC = Akaike information criterion; BIC = Bayesian information criterion; TLI = Tucker Lewis index; CFI = confirmatory fit index; RMSEA = root mean square error of approximation; SRMR = standardized root mean square residual.

**Table S2.** Descriptive statistics and internal consistency of the BNSS (N=912)

| BNSS domain, subscale, item, and total scores | Mean | SD | Cronbach's alpha |
| --- | --- | --- | --- |
| Motivational Deficit | 20.62 | 9.7 | 0.94 |
| *Anhedonia subscale* | 8.61 | 4.58 | 0.96 |
| 1. Intensity of pleasure | 2.85 | 1.57 |  |
| 2. Frequency of pleasure | 2.95 | 1.59 |  |
| 3. Intensity of expected pleasure | 2.82 | 1.62 |  |
| *Asociality subscale* | 6.33 | 3.03 | 0.88 |
| 5. Asociality:behavior | 3.3 | 1.6 |  |
| 6. Asociality: internal experience | 3.03 | 1.61 |  |
| *Avolition subscale* | 5.68 | 3.16 | 0.92 |
| 7. Avolition: behavior | 2.88 | 1.66 |  |
| 8. Avolition: internal experience | 2.80 | 1.62 |  |
| Expressive Deficit | 12.85 | 8.08 | 0.95 |
| *Blunted affect subscale* | 8.06 | 5.07 | 0.96 |
| 9. Facial expression | 2.72 | 1.7 |  |
| 10. Vocal expression | 2.64 | 1.8 |  |
| 11. Expressive gestures | 2.7 | 1.79 |  |
| *Alogia subscale* | 4.79 | 3.51 | 0.94 |
| 12. Quantity of speech | 2.26 | 1.77 |  |
| 13. Spontaneous elaboration | 2.53 | 1.84 |  |
| BNSS Total score | 35.91 | 17.55 | 0.96 |
| BNSS total score excluding distress | 33.47 | 16.59 | 0.96 |

N= number of participants; SD= standard deviation; BNSS= Brief Negative Symptom Scale

**2.** **Internal consistency of the Brief Negative Symptom Scale (BNSS) in a large European, multicenter study**

**2.1 Methods**

The data set used to evaluate the internal consistency of the Brief Negative Symptom Scale in the current investigation was that included in the study of Mucci et al., 2019 [3].

The internal consistency of the BNSS, its subscales and factors was assessed using the Cronbach’s alpha value. All the analyses were carried out with SPSS Statistics (version 28).

**2.2 Results**

Results regarding the BNSS internal consistency are reported in table S3.

The 13-item BNSS had good internal consistency (Cronbach's alpha = 0.94) and there were no changes in Cronbach's alpha after removing Distress. Cronbach's alpha values of sub-scales ranged from 0.8 to 0.93. For MAP and EXP factors, Cronbach's alpha was 0.93.

**Table S3.** Descriptive statistics and internal consistency of the BNSS

| BNSS domain, subscale, item, and total scores | N | Mean | SD | Cronbach's alpha |
| --- | --- | --- | --- | --- |
| Motivational Deficit | 248 | 15.11 | 9.2 | 0.93 |
| *Anhedonia subscale* | 248 | 6.31 | 4.67 | 0.92 |
| 1. Intensity of pleasure | 249 | 2.1 | 1.61 |  |
| 2. Frequency of pleasure | 248 | 2.1 | 1.64 |  |
| 3. Intensity of expected pleasure | 249 | 2.1 | 1.78 |  |
| *Asociality subscale* | 249 | 4.2 | 2.58 | 0.8 |
| 5. Asociality:behavior | 249 | 2.33 | 1.54 |  |
| 6. Asociality: internal experience | 249 | 1.87 | 1.3 |  |
| *Avolition subscale* | 249 | 4.58 | 3 | 0.88 |
| 7. Avolition: behavior | 249 | 2.41 | 1.58 |  |
| 8. Avolition: internal experience | 249 | 2.2 | 1.59 |  |
| Expressive Deficit | 249 | 9.9 | 6.97 | 0.93 |
| *Blunted affect subscale* | 249 | 6.71 | 4.43 | 0.93 |
| 9. Facial expression | 249 | 2.37 | 1.5 |  |
| 10. Vocal expression | 249 | 2.06 | 1.62 |  |
| 11. Expressive gestures | 249 | 2.28 | 1.6 |  |
| *Alogia subscale* | 249 | 3.2 | 3.09 | 0.92 |
| 12. Quantity of speech | 249 | 1.35 | 1.45 |  |
| 13. Spontaneous elaboration | 249 | 1.84 | 1.76 |  |
| BNSS Total score | 246 | 26.5 | 15.5 | 0.94 |
| BNSS total score excluding distress | 248 | 25.03 | 14.65 | 0.94 |

N= number of participants; SD= standard deviation; BNSS= Brief Negative Symptom Scale

**References**

1. Mucci A, Galderisi S, Merlotti E, et al. The Brief Negative Symptom Scale (BNSS): Independent validation in a large sample of Italian patients with schizophrenia. Eur Psychiatry. 2015;30(5):641-647. doi:10.1016/j.eurpsy.2015.01.014
2. Galderisi S, Mucci A, Dollfus S, et al. EPA guidance on assessment of negative symptoms in schizophrenia. Eur Psychiatry. 2021;64(1):e23. doi:10.1192/j.eurpsy.2021.11
3. Mucci A, Vignapiano A, Bitter I, et al. A large European, multicenter, multinational validation study of the Brief Negative Symptom Scale. Eur Neuropsychopharmacol. 2019;29(8):947-959. doi:10.1016/j.euroneuro.2019.05.006
